# Online Detection of P300 and Error Potentials in a BCI Speller

**DOI:** 10.1155/2010/307254

**Published:** 2010-02-11

**Authors:** Bernardo Dal Seno, Matteo Matteucci, Luca Mainardi

**Affiliations:** ^1^IIT-Unit, Department of Electronics and Information, Politecnico di Milano, Piazza Leonardo da Vinci 32, 20133 Milano, Italy; ^2^IIT-Unit, Department of Bioengineering, Politecnico di Milano, Piazza Leonardo da Vinci 32, 20133 Milano, Italy

## Abstract

Error potentials (ErrPs), that is, alterations of the EEG traces related to the subject perception of erroneous responses, have been suggested to be an elegant way to recognize misinterpreted commands in brain-computer interface (BCI) systems. We implemented a P300-based BCI speller that uses a genetic algorithm (GA) to detect P300s, and added an automatic error-correction system (ECS) based on the single-sweep detection of ErrPs. The developed system was tested on-line on three subjects and here we report preliminary results. In two out of three subjects, the GA provided a good performance in detecting P300 (90% and 60% accuracy with 5 repetitions), and it was possible to detect ErrP with an accuracy (roughly 60%) well above the chance level. In our knowledge, this is the first time that ErrP detection is performed on-line in a P300-based BCI. Preliminary results are encouraging, but further refinements are needed to improve performances.

## 1. Introduction

A brain-computer interface (BCI) is an interface that does not entail muscle movements, but it bypasses any muscle or nerve mediation and connects a computer directly with the brain by picking up signals generated by the brain activity.

Among the different kinds of brain activity that can be used in a BCI, the *P300* phenomenon has been known [1] and investigated for many years. It is an event-related potential (ERP), traditionally described as a positive peak visible in an EEG recording at approximately 300 ms from an event. It follows unexpected, rare, or particularly informative stimuli, and it is typically stronger in the parietal area. The shape of the P300 depends on the characteristics of the stimuli and their presentation.

For BCI applications, the “exact” shape of the P300 is not so important as having a way to detect it. Detecting a P300 in a single trial is very difficult and, therefore, repeated stimuli are normally used to facilitate the selection of the one that has generated a P300. The number of repetitions can be predetermined for each user to get the best trade-off between speed and accuracy.

**Figure 1:**
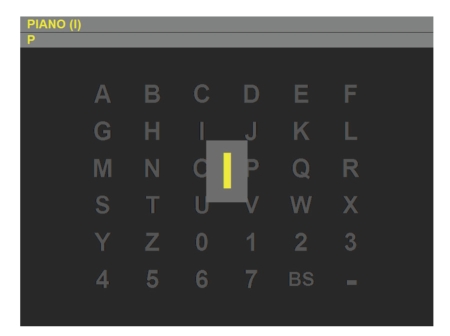
Graphical interfaces of the P300 spellers used in the experiments, showing the moment of the letter feedback used for ErrP-based confirmation.

In [2], Donchin and colleagues presented the first P300-based BCI, called also P300 speller, which permits to spell words. A grid of letters and symbols is presented to the user, and entire columns or rows are flashed one after the other in random order (see Figure 1 for an example). When the column/row containing the desired letter is flashed, a P300 is elicited. In Donchin's work, classification is made through stepwise discriminant analysis (SWDA) applied to averages of samples from epochs relative to the same stimulation (same row or same column).

Other BCI interfaces using the P300 protocol have been developed since then. In [3], a virtual-reality system is presented where subjects operate objects selected through the P300. Classification is made by comparing the correlation of single responses with the averages of all target and nontarget responses. In [4], subjects (healthy and impaired ones) control a cursor by choosing among four commands (up, down, left, right) via the P300. In this case, single-sweep detection is performed: independent component analysis (ICA) is used to decompose the EEG signal, a fuzzy classifier identifies a candidate P300 component among the ones extracted by ICA, and a neural network classifies it as target or nontarget. The system is more effective with healthy subjects, though no exact reason could be pinpointed. Finally, in [5], an initial attempt at using a BCI in a home environment is reported: a person with amyotrophic lateral sclerosis uses a P300 speller on a daily basis.

Another relevant event-related potential is the *error potential* (ErrP hereafter), which is generated when a subject makes a mistake, and, more interestingly for BCI applications, when the machine behaves differently from the user intent. Known since the late 1980s [6, 7], ErrPs were described as a negative shift in the electric potential over the fronto-central region (from Fz to Cz of the 10–20 system) occurring 50–100 ms after an erroneous response (*error negativity*—Ne or *error-related negativity*—ERN) and a subsequent positive shift in the parietal region, whose maximum occurs between 200 and 500 ms after the error (*error positivity*—Pe). A high variability in shape, size, and delay of the Ne and Pe components has been observed as the effect of different underlying mechanism, whose nature is not yet certain [8].

In [9] the presence of ErrPs in a BCI paradigm (cursor movement by mu and beta rhythms) was revealed, as a positive peak at Cz 40 ms after the end of erroneous trials. This finding suggests an interesting application: the automatic detection of the errors made by a BCI in recognizing the user's intent and a way to improve its performances. Millán and colleagues [10, 11] made experiments with ErrPs found in a motor-imagery BCI. They trained a Gaussian classifier to automatically recognize ErrPs, reaching an accuracy of about 80%.

 In this work we present our experience in detecting P300 and ErrP in a P300-based speller with an integrated automatic error-correction system (ECS) based on the single-sweep ErrP detection.

## 2. Experimental Setting

We developed a classical BCI based on P300, the P300 speller, and integrated the use of ErrP in it. Our P300 speller is very similar in appearance and in functioning to the paradigm described by Donchin et al. [2]: 36 symbols are disposed on a 6 × 6 grid, and entire rows and columns of symbols are flashed one after the other in random order. The grid of symbols is visible in Figure 1. There are the letters from the alphabet, some digits, the space, and the *backspace*, represented as BS in the right bottom corner. The intensification of rows and columns lasts for 125 ms and the matrix remains blank for 125 ms between two consecutive flashes. Each row and column is flashed exactly once in the first 12 stimulations (a block of 12 consecutive stimulations is called a *repetition*); then another round of 12 stimulations is repeated, with flashing of rows and columns done in a new random order. We used 5 repetitions with no pause between repetitions. Please note that the number of repetitions is lower than usual. The choice is instrumental to stress the system under an unfavorable situation, thus soliciting a substantial number of misspelled letters.

After the fifth repetition, the P300 system detects the row and the column that are more likely to have elicited a P300, and selects the letter at their intersection. After a pause of 1 s, the letter is presented to the user in a big rectangle that pops up in front of the grid (see Figure 1). The presentation of the letter should elicit an ErrP if the letter predicted by the P300 system is different from the one the user intended.

An ErrP detection system figures out if any ErrP is elicited by the presentation of the selected letter, and in that case it overrides the P300 speller and cancels the last selection; otherwise, the letter is appended to the text at the top. After a 2–3 s pause (this parameter is tuned to each subject's requirements), the speller starts a new series of stimulations for the next letter. A *trial*, in this context, is the whole series of 60 row/column flashes (12 flashes times 5 repetitions) together with the feedback of the speller selection made for each letter, that is, a single trial is composed of 60 P300 stimulations and 1 ErrP stimulation (a trial is about 15 s long).

In the online experiments, the users interact with the speller in two ways: in *copy mode* they are asked to select letters indicated by the BCI before each trial, so as to simplify the evaluation of the performance; in *free mode* subjects spell words of their own choosing. In *copy mode*, the system performs one trial for each letter, and it goes on to the next letter even when the P300 classifier is wrong; in this mode the ErrP correction system is not active. In *free mode* the ErrP correction system is active, and the user has to hit the *backspace* to correct a misspelled letter only when the error is not automatically recognized. During training, the speller is used in *copy mode* only. The GA and ErrP training was performed sequentially. In the ErrP training, in order to elicit error-related responses, the letter feedback is chosen wrongly in 20% of the times and correctly in 80% of the cases.

 The speller we have implemented is based on BCI2000 [12], a general-purpose software system developed at the Wadsworth Center of the New York State Department of Health in Albany, New York, USA, for brain-computer interface (BCI) research. We developed three main components: a source module that acquires EEG data from our amplifier, an application derived from the built-in P300 speller, and a dual-classifier processing module to handle both P300 and ErrP classification. The application module implements the P300 speller with ErrP-based error correction, as described above, and a precise synchronization system (fully described in [13]). The processing module splits the EEG signals in epochs synchronized on the stimulation instants, processes the data, and performs the classification of the epochs according to two separate processing chains, one for P300s and one for ErrPs, briefly described below.

## 3. Data Processing

EEG data are acquired with an EBNeuro BE Light amplifier at locations Fz, Cz, Pz, and Oz, and at a frequency of 512 Hz. Also, EOG is recorded from the right eye of the subject. EOG is not used for classification, but it is used to discard noisy epochs during training and is kept for future analysis. For P300 detection, a logistic classifier [14] is used, trained on features extracted through a genetic algorithm.

Genetic algorithms are a class of optimization algorithms that mimic the way natural evolution works. In a genetic algorithm, a set of possible solutions to an optimization problem are coded in strings called *chromosomes*; solutions are evaluated, and the best ones (those with highest *fitness*) are selected and combined together to form new possible solutions, in a process that mimics evolution among living beings. After some repetitions of the procedure, good solutions emerge.

**Figure 2:**
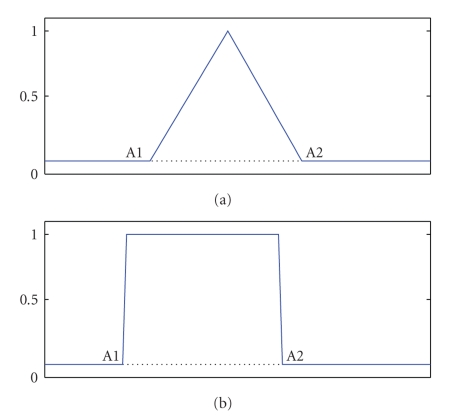
Weight functions used for feature extraction.

In the genetic algorithm used in this work, each individual (chromosome) represents a set of possible features for discriminating the presence of a P300 in EEG recordings. Each gene encodes a feature and an EEG channel from which to extract it; a feature is obtained by multiplying the EEG channel by a weight function, whose exact shape is encoded by parameters in genes (see Figure 2 for examples of weight functions). Genetic operators are a variant of 1-point crossover and uniform mutation, and tournament selection with elitism is used. The fitness of a chromosome is the 4-fold cross-validated performance obtained by training a logistic classifier on the encoded features extracted from the training set. For a complete description of the algorithm, please see [15].

An analysis of the combination of the features extracted by the genetic algorithm and the classifier trained on the training set allows to compute weights assigned to individual EEG samples. In this way, the resulting classifier is very fast to apply online.

For ErrP detection, a simpler method is used, also because fewer training data are available (there is one ErrP stimulation per letter versus sixty for P300).

EEG data are segmented in epochs, whose extremes are found with the algorithm explained below. Epochs are then filtered in the band 1–10 Hz to improve the signal-to-noise ratio by eliminating frequency components extraneous to ErrPs. EEG samples are fed into a classifier trained through linear discriminant analysis (LDA).

On average, a difference between ErrP and non-ErrP epochs is observable only in some intervals of the segmented epoch, and these intervals depend on the subject. For these reasons we developed a way to automatically determine significant intervals in the ErrP for classification from the training data. Training data are first segmented in epochs ranging from 100 ms before the stimulation instant (feedback onset) to 500 ms after it. Epochs containing strong EOG activity (>100 *μ*V at any point) are automatically discarded before further analysis. A 1–10 Hz pass-band filter is then applied. For each channel *c* and time point *t*, the signals *s*_*c*,1_(*t*) from ErrP epochs and *s*_*c*,0_(*t*) from non-ErrP epochs can be viewed as two sets of random variables. A *t*-test is used to check if, for any given *t* and *c*, the mean of *s*_*c*,1_(*t*) differs significantly from the mean of *s*_*c*,0_(*t*); the significance level has been chosen to be 0.01, but much smaller *P*-values have been often found in analyzing data. The *t*-test is used only to find a time interval to use for classification, so the validity of its assumptions (Gaussian distributions with equal variance) is not very important; nevertheless, we ran some statistical tests on the data and they were satisfied.

**Figure 3:**
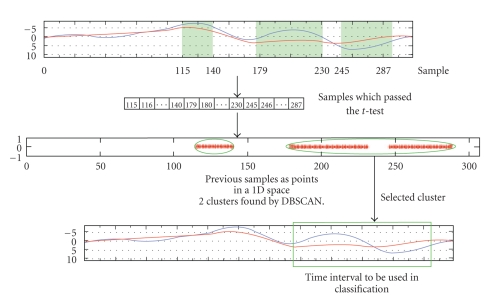
Procedure for the identification of significant intervals. Top: shadowed areas contain the samples that passed the *t*-test with a *P*-value of  .01 or less. Middle: clustering of samples. Bottom: the interval used for classification.

The points detected by the *t*-test tend to lie in groups, because the filtered signals have a strong autocorrelation for short lags. However, many intervals of different sizes, with “holes” in between (see the top part of Figure 3 for an example), are usually detected, while we are interested only in finding one contiguous time interval containing all the interesting features of signals. We used DBSCAN [16], a clustering algorithm based on density, to fill holes and discard isolated points or small intervals. The biggest interval is selected and used for classification.

The training phase produces both a time interval and a linear classifier. During online classification, the procedure is very fast. EEG epochs are cut according to the interval found, and the classifier is applied to pass-band filtered epochs.

## 4. Results

Three subjects participated in a first set of online experiments. The P300 speller used 5 repetitions of each stimulation per letter; for Subject B1 we had to reduce the number of repetitions to 4 in order to have a reasonable number of errors (the online performance in *copy mode* for Subject B1 was 90% with 5 repetitions). On the other hand, Subject B2 had a poor performance mainly due to low concentration; the subject reported problems in focusing on the task, probably because of a failure of the brightness regulation of the computer screen that affected the online recordings. All results confirm the offline figures reported in [15], and confirm the validity of the GA-based classification method.

Subjects B1 and B3 also used the BCI to spell words in *free mode*, where the error correction mechanism was enabled. The results are shown in Table 1 and confirm that the classifier found by the GA can be used to really drive a BCI application. Subject B2 could have tried to use the speller by increasing the number of repetitions, but as the data was recorded also to evaluate error potentials, this would have made the recording sessions much longer.

Results of the online experiments are shown in Table 2. The classifiers were tested in sessions different from those used for training, so they are really indicative of a possible online use. For both users the classification performance is well above chance level, but this is not enough to say whether ErrP detection has been useful for such users. To test it, we computed the *gain* obtained by the inclusion of an automatic ErrP correction system. The gain is based on the computation of the *Utility* metric we recently proposed (see [17] for details): for subject B1 we obtained a small improvement (gain = 1.0011), while in subject B3 a detriment to the performance is observed (gain = 0.8733).

## 5. Discussion

In this paper we have presented an experiment that—to the best of our knowledge—is the first attempt to use a P300 BCI with an integrated error-correction mechanism based on ErrPs. Although the number of subjects participating to the online study is quite limited, results are encouraging and confirm the feasibility of ErrP single-sweep detection already verified in more populated offline studies such as [18] or [13].

The use of a genetic algorithm for the definition of features to be used in P300 detection has proven its strength also in the online use, after good results in offline analysis [15]. In principle, the very same algorithm could be used for the ErrP feature design, but this is somehow prevented by the reduced number of examples that can be gathered in training sessions. A different strategy could be devised to collect ErrP examples automatically during the use of our P300-based BCI application. Each back space in free mode can be treated as an explicit tagging of an ErrP by the user. With this strategy, data gathering would be still time consuming (we are not changing the odds for ErrP elicitation after all), but it could be more acceptable by the user, and it might enhance her experience with the speller as time passes.

The results presented are encouraging, but some additional work is still needed to improve the performance. In particular, it is important that ErrP detection reaches a high accuracy, higher than P300 detection. The reason is that ErrP stimulations are generated only once after each letter selection, and this is the only chance to detect an ErrP. An accuracy higher than chance is not sufficient to have a usable interface or a significant improvement as documented by the measured gain. In addition, to make the inclusion of ErrP corrections more profitable, the performances of the ErrP classifier should be tuned on a user-by-user basis. This could be done by maximizing the above mentioned gain. This was not done in the present paper, where we did not tune the ErrP classifier in favor of false positives (nor false negatives [19]).

In addition, to make the system more useful in practice, we plan to refine our processing methods and presentation interface (to better capture the subject attention) so as to increase the performance of the ErrP classifier; in a more extensive study with more subjects we will assess the online performance of the enhancement.

## Figures and Tables

**Table 1:** Results of the GA online in *free mode*. Training set size is the number of letters spelled in *copy mode* to collect training examples for the GA classifier. Performance is given as the number of correctly predicted letters over the total numbers of letters in the online usage.

Subject	Training	No.	online
	set size	repetitions	performance
B1	196	4	74/109 (68%)
B3	108	5	137/202 (68%)

**Table 2:** Results of the online ErrP classification. Training size is the number of letters for each class from the ErrP *copy mode* session. Performance is the fraction of correct classification in the *free mode* experiment.

		Train.	online
Subject		Size	performance
B1	ErrP	84	23/35 (66%)
	N-ErrP	290	51/74 (69%)
B3	ErrP	65	38/65 (58%)
	N-ErrP	193	91/137 (66%)
